# IL28B polymorphism, Explanation for Different Responses to Therapy in Hepatitis C Patients

**DOI:** 10.5812/kowsar.1735143X.794

**Published:** 2011-12-20

**Authors:** Heidar Sharafi, Seyed Moayed Alavian

**Affiliations:** 1 Armin Pathobiology Laboratory, Tehran, IR Iran; 2 Baqiyatallah Research Center for Gastroenterology and Liver Diseases, Baqiyatallah University of Medical Sciences, Tehran, IR Iran

**Keywords:** Polymorphism, Genetic, Hepatitis C, IL28B Protein, Human

Hepatitis C infection is a major global public health problem [1]. Treatment of hepatitis C with pegylated interferon α plus Ribavirin as a standard of care (SOC) for the management of this disease, leads to eradication of the virus in less than 60% of patients [2]. Different sustained virological response (SVR) rates in different populations is a challenging fact that has been observed by researchers and inspired them to search for the causes [3]. In September 2009, Ge et al. [4] in a genome-wide association study (GWAS) found the rs12979860 single nucleotide polymorphism (SNP), which is located 3 kb upstream of the IL28B gene, to be the strongest host genetic predictor of SVR in hepatitis C genotype 1. They observed that rs12979860 CC patients, regardless of their ethnicity, reach SVR rates approximately twice that of rs12979860 TT patients. In less than a 3 year time span, from the first report by Ge et al. till the present (December 2011), around 250 papers on the association of IL28B SNP with hepatitis C outcomes have been indexed in Medline/Pubmed, which is a singular reflection of the high importance this topic has for medical researchers. Also, Ge et al. found a different frequency of the IL28B rs12979860, with the highest favorable allele in Asians and the lowest in Africans, which in part elucidates the high rate of SVR in Asians and the low rate of SVR in Africans. Later in 2009, two GWAS by Suppiah et al. [5] and Tanaka et al. [6] found rs8099917, another IL28B polymorphism which is located around 8 kb upstream of the IL28B gene, to be the strongest genetic determinant of SVR in hepatitis C virus (HCV) genotype 1 infected patients. Here we should note that:

1) These two SNPs, rs12979860 and rs8099917, are in partial linkage disequilibrium (LD) in Caucasians [4][7] and

2) Although a few studies such as a meta-analysis by Li et al. [8] showed that rs12979860 can predict SVR better than rs8099917, further studies are needed to assess the exact role and impact of each of these two SNPs on hepatitis C outcomes in different populations. Initially, most of the studies concentrated on the HCV genotype 1, which is the most frequent type in Western countries, and observed the same association between IL28B SNP and SVR that was found by Ge et al. On the other hand, the impact of IL28B polymorphisms on the outcome of HCV genotypes non-1 such as HCV genotype 4 [9][10] and HCV genotype 2 or 3 [7][11][12][13] has been studied as well. Unlike the results of studies that investigated the impact of IL28B SNP on SVR in HCV genotype 1 infected patients, results of studies using HCV genotype 2 or 3 infected patients are not similar and some found no association between IL28B SNP and SVR to standard of care in HCV genotype 2 or 3 infected patients [7][12], while some found such an association [11][13]. SVR as a hepatitis C treatment outcome can be predicted by various baseline predictors such as HCV RNA levels, the dose and duration of therapy, body mass index, age, insulin resistance, gender, stage of fibrosis and co-infection with other hepatitis viruses or HIV [14]. Also, we know that the viral kinetic which is assessed by the rapid virological response (RVR) can predict SVR stronger than baseline predictors that have been identified above. In 2009, after the discovery of IL28B SNP as the strongest host genetic determinant of SVR, we assumed that IL28B SNP could be an alternative to RVR, but according to our experience it seems that RVR is a reflection of all known and unknown predictors of treatment outcome and cannot be replaced by IL28B SNP.

In 2011, the European Association for the Study of the Liver (EASL) and the American Association for the Study of Liver Diseases (AASLD) included IL28B testing in their guidelines [14][15]. Here we recommend that physicians consider IL28B genotyping in the sub-groups of patients with indications for IL28B testing according to EASL and/or AASLD guidelines. Also, relapsers and non-responders with favorable pre-treatment predictors compel us to investigate and discover new predictors that can explain treatment failure in such patients. Sporea et al. [16] studied the correlation of IL28B rs12979860 with SVR in Romanian patients with chronic hepatitis C treated with a SOC regimen and subsequently observed SVR rates approximately 30% higher in rs12979860 CC patients than in non-CC patients. We noticed that in their study, IL28B rs12979860 genotype distribution was not in the Hardy-Weinberg equilibrium (HWE). Since the deviation from HWE can be a result of genotyping error, researchers should test the genotyping data for deviation from HWE and should consider it in their reports [17]. Finally, we report that the finding of the correlation between IL28B SNP and hepatitis C outcomes, in part clarified the inter-individual variation in treatment responses observed in the clinic. IL28B SNP as a pharmacogenetic marker of IFN-based treatments can be considered in treatment decision making.
